# Exploring the mechanism of Yi Qi Huo Xue compound prescription in treating chronic heart failure based on integrated network pharmacology and transcriptomics

**DOI:** 10.3389/fcvm.2026.1738535

**Published:** 2026-03-23

**Authors:** Jiadan Liao, Pengcheng Wang, Zuoyue Wu, Juan Cui, Peiyun Shen, Xufeng Chen

**Affiliations:** 1 Department of Cardiology, The Third Afﬁliated Hospital of Zhejiang Chinese Medical University, Hangzhou, China; 2 Department of Tuberculosis, Hangzhou Red Cross Hospital, Hangzhou, China; 3 Department of Cardiology, Longquan People’s Hospital, Lishui, China; 4 Department of Cardiology, Tongde Hospital of Zhejiang Province, Hangzhou, Zhejiang, China

**Keywords:** chronic heart failure, key target genes, network pharmacology, transcriptomics, Yi Qi Huo Xue compound prescription

## Abstract

**Background:**

Chronic heart failure (CHF) represents a major global health burden characterized by complex pathologies. The Yi Qi Huo Xue compound prescription (YQHXCP) has demonstrated significant clinical efficacy in alleviating heart failure symptoms; however, its precise molecular mechanisms remain obscure.

**Objective:**

This study aims to elucidate the core targets and biological pathways of YQHXCP in treating CHF through an integrated approach combining network pharmacology with transcriptomic validation.

**Methods:**

A rat model of heart failure was established, and transcriptomic data were acquired via RNA sequencing. Concurrently, putative targets of YQHXCP were retrieved from the TCMSP and SwissTargetPrediction databases. Key targets were identified by intersecting differentially expressed genes (DEGs) from the animal model with predicted drug targets. Subsequently, functional enrichment analysis, gene-gene interaction (GGI) network construction, and molecular docking were employed to decipher the underlying mechanisms, followed by *in vivo* validation using RT-qPCR.

**Results:**

Three pivotal target genes were identified: Top2a, Cdk1, and E2f2. Enrichment analysis revealed that YQHXCP primarily modulates mitochondrial protein complexes, ribosomal subunit assembly, and cell cycle checkpoints. Molecular docking demonstrated strong binding affinity between the active ingredient quercetin and both Cdk1 and E2f2 proteins. RT-qPCR confirmed that YQHXCP significantly reversed the downregulation of Cdk1 and E2f2 expression in the myocardial tissue of CHF rats.

**Conclusion:**

YQHXCP may exert its anti-CHF effects by targeting Cdk1 and E2f2 to regulate mitochondrial function and cell cycle homeostasis. These findings provide novel insights into the multi-target therapeutic mechanisms of traditional Chinese medicine formulations.

## 1 Introduction

Chronic heart failure (CHF) is a complex clinical syndrome characterized by impaired ventricular filling or ejection capacity due to cardiac structural or functional abnormalities, resulting in inadequate tissue perfusion and pulmonary congestion (1). This condition has become a global public health challenge, with persistently high prevalence and mortality rates. Epidemiological data indicate that the number of patients with heart failure worldwide exceeds 64 million, and according to the latest report from the American Heart Association (AHA), the prevalence of heart failure is projected to increase by 46% by 2030 (2, 3) The primary clinical manifestations of CHF include dyspnea, fatigue, and fluid retention (e.g., lower extremity edema), severely impairing quality of life. Frequent hospitalizations impose a substantial economic burden on healthcare systems (4, 5). Currently, guideline-directed medical therapy (GDMT), including renin-angiotensin system inhibitors, β-blockers, and mineralocorticoid receptor antagonists, serves as the cornerstone of CHF management. Although these therapies can delay disease progression and improve prognosis to some extent, they have significant limitations (6). On one hand, some patients cannot tolerate target doses due to adverse effects such as hypotension, bradycardia, worsening renal function, and electrolyte disturbances (7). On the other hand, despite receiving standardized treatment, a considerable proportion of patients exhibit suboptimal responses, with ongoing disease progression. Therefore, in-depth exploration of the molecular mechanisms underlying the onset and progression of CHF is of critical importance for identifying novel therapeutic targets, developing safer and more effective drugs, accurately predicting patient prognosis, and formulating individualized treatment strategies.

The Yi Qi Huo Xue compound prescription (YQHXCP) is an empirically formulated experience-based formula developed by the authors, targeting the pathogenesis of heart failure characterized by deficiency of qi as the root, and blood stasis, phlegm-turbidity, and fluid retention as the manifestations. Guided by the therapeutic principle of reinforcing qi, activating blood circulation, and promoting diuresis, this formula consists of Huangqi, Renshen, Fuling, Danshen, Yimucao, Tinglizi, Honghua, and Sanqi (8). Clinically, it has demonstrated excellent performance in treating CHF, particularly in alleviating symptoms and enhancing efficacy while reducing toxicity when combined with Western medicine (9). However, the pathways and molecular targets through which its complex components exert their effects require more comprehensive and in-depth investigation.

Network pharmacology is an emerging interdisciplinary field that systematically elucidates the synergistic mechanisms of drugs through multi-target interactions by constructing multi-layered networks of “drug-component-target-disease” (10). Its core advantage lies in breaking away from the traditional “one drug-one target” research paradigm, which aligns well with the therapeutic characteristics of traditional Chinese medicine (TCM) formulations—featuring multiple components, multiple targets, and holistic regulation—thus providing a powerful tool for scientifically interpreting the complex mechanisms of TCM (11). Currently, network pharmacology has been widely applied in research on cancer, metabolic diseases, and cardiovascular disorders. For instance, in the field of cardiovascular disease, this approach has successfully uncovered potential targets and signaling pathways through which single herbs such as Salvia miltiorrhiza (Danshen) and Astragalus membranaceus (Huangqi), as well as their compound formulas, regulate pathological processes like myocardial hypertrophy and atherosclerosis (12, 13). These findings preliminarily suggest that their effects may involve multiple biological processes, including anti-inflammation, antioxidation, inhibition of myocardial fibrosis, and regulation of cell apoptosis and autophagy (14, 15). These discoveries offer valuable insights into the molecular basis of TCM in treating heart failure.

However, despite its established clinical efficacy, the specific molecular targets and synergistic mechanisms through which the complex components of the Yi Qi Huo Xue compound prescription (YQHXCP) exert their effects remain largely elusive, representing a significant gap in the current literature. To address this, we employed an integrated strategy combining network pharmacology and *in vivo* cardiac transcriptomics to bridge the gap between theoretical targets and actual pathological changes. Network pharmacology offers a systematic approach to predict the holistic scope of potential targets based on chemical composition, which breaks away from the traditional “one drug-one target” paradigm and aligns perfectly with the multi-component, holistic regulatory nature of traditional Chinese medicine (TCM). Meanwhile, transcriptomics captures the specific gene expression alterations induced by the disease state, providing crucial *in vivo* experimental validation for these computational predictions. By intersecting these two datasets from a rat chronic heart failure (CHF) model, we aim to filter out non-specific targets and pinpoint core genes that are both theoretically druggable by YQHXCP and pathologically relevant to CHF progression. Based on the traditional therapeutic principle of “reinforcing qi and activating blood”, we hypothesize that YQHXCP mitigates CHF and reverses pathological remodeling by modulating specific gene networks associated with energy metabolism and cell cycle regulation. Ultimately, this study aims to systematically elucidate the core target genes and biological signaling pathways underlying YQHXCP therapy.

## 2 Materials and methods

### 2.1 Building the rat model

**Figure 1:**
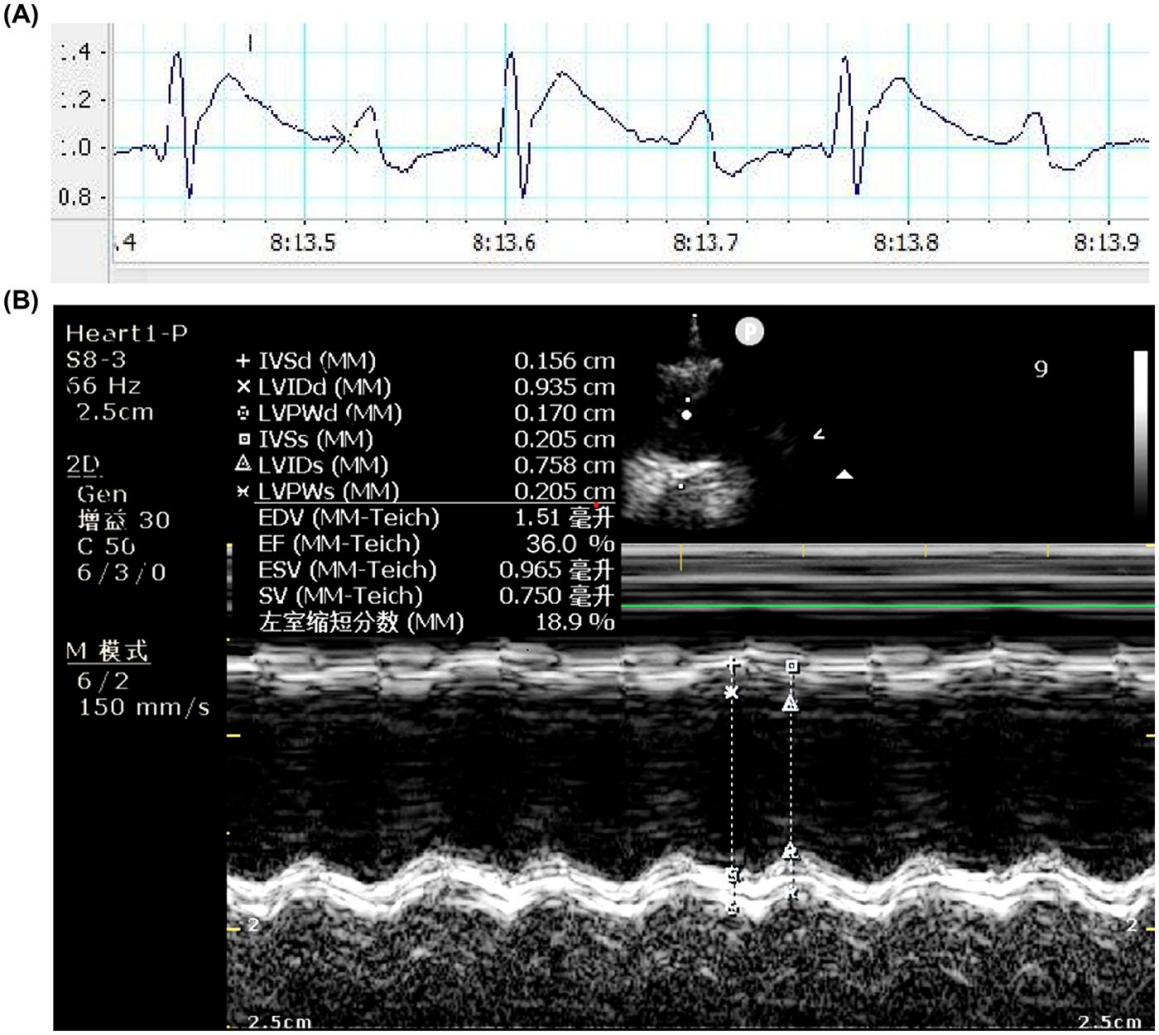
Establishment and validation of the chronic heart failure (CHF) rat model. **(A)** Representative electrocardiogram demonstrating ST-segment elevation immediately following coronary artery ligation. **(B)** Representative echocardiography acquired 4 weeks post-surgery. The image displays quantitative measurements, with an EF ≤ 40% utilized as the indicator of successful heart failure modeling.

SPF-grade male SD rats aged 6 to 8 weeks were selected, which were acquired from Beijing Sibeifu Bio-Technology Co., Ltd. [Production License No.:SCXK (Beijing) 2019-0010;Use License No.:SYXK (Dian) K2022-0006]. The rats was placed in a supine position. The skin over the left chest, from the sternum to the anterior axillary line between the 2nd and 5th ribs, was prepared. Local disinfection was performed with iodophor, and a disposable sterile drape was placed. After the skin was incised, the pectoralis major and serratus anterior muscles were bluntly separated layer by layer to expose the ribs. Using a curved hemostat, the intercostal muscles and pleura at the 3rd–4th intercostal space were gently punctured to expose the heart. A curved forceps was used to puncture the pericardium, and a cardiac retractor was inserted to gently lift the heart, revealing the left auricle and pulmonary artery cone. Ligation was performed using a 6–0 suture approximately 2 mm below the root of the left auricle at the origin of the left main coronary artery, with a puncture depth of about 0.5–1 mm and width of about 1–2 mm. After ligation, the heart was carefully repositioned back into the thoracic cavity, and the sutured cotton ball was pulled out. Elevation of the ST segment on the electrocardiogram was observed (Figure 1A). Four weeks after surgery, combined with exhaustive swimming and reduced food intake, the left ventricular ejection fraction (EF value) was measured by Doppler echocardiography and found to be ≤40% (Figure 1B), which was used as an indicator of successful heart failure modeling. At the same time, the rats exhibited weight loss, lethargy, and dull, lusterless fur, consistent with the Traditional Chinese Medicine (TCM) pattern diagnosis of qi deficiency and blood stasis.

The rats were randomly split into four groups and corresponding drug interventions were administered: heart failure group (C group), YQHXCP group (K group), lisinopril group (L group) and the sham group (I group). The I group underwent needle insertion and threading beneath the root of the left auricle without ligation, while all other procedures were identical to those in the surgical groups.

### 2.2 Drug composition and administration dosage

The YQHXCP consists of Astragalus membranaceus (Huangqi) 25 g, Panax ginseng (Renshen) 20 g, Poria cocos (Fuling) 25 g, Salvia miltiorrhiza (Danshen) 30 g, Leonurus japonicus (Yimucao) 30 g, Descurainia sophia (Tinglizi) 15 g, Carthamus tinctorius (Honghua) 15 g, and Panax notoginseng (Sanqi) 3 g. All raw herbal materials were purchased from Huadong Medicine Co., Ltd. and authenticated by experienced pharmacists according to the Pharmacopoeia of the People's Republic of China (2020 Edition). Detailed quality control was performed using High-Performance Liquid Chromatography (HPLC) to ensure the stability of key components. Water-soluble granules were prepared fresh before each use to ensure therapeutic efficacy. Lisinopril tablets were produced by Shantou Jinshi Pharmaceutical General Factory, with each tablet containing 5 mg (National Drug Approval Number: H20065767).

Dosages were calculated based on the “Equivalent Dose Conversion Table for Body Surface Area between Humans and Animals” from Pharmacological Experimental Methods (18th edition). Using the conversion coefficient of 0.0026 between humans (70 kg) and rats (200 g), the daily crude drug dose for a 200 g rat was calculated as 3.9 g, equivalent to 19.5 g/kg. The K group received the YQHXCP via oral gavage at a daily dose of 540 mg/kg. The L group received lisinopril via oral gavage once daily. The C group and I group received an equivalent volume of sterile water for injection via gavage. All treatments were administered for 12 weeks.

### 2.3 Sample collection

After 12 weeks of continuous administration, rats were euthanized under anesthesia induced by 20% urethane. Subsequently, cardiac tissues were collected from rats in the I group, C group, K group, and L group, respectively.

### 2.4 TTC staining

Immediately after extraction, the hearts were submerged in a PBS solution maintained at a temperature range of 0–4°C. After a short period in the PBS bath, they were transferred to a −20°C freezer for a 30 min freezing session. Subsequently, using a precision cutting device, the frozen hearts were sliced into segments with a thickness of precisely 2 mm. These heart slices were then carefully placed in a 2% red tetrazolium staining solution. To ensure accurate staining results, the solution containing the slices was placed in a 37°C water bath, and the setup was shielded from any light source. During the 30 min staining duration, the containers were gently agitated every five minutes to facilitate uniform staining of the heart slices.

Upon completion of the staining process, the heart slices were carefully removed from the staining solution and rinsed with a PBS solution for a period of 3 to 5 min. Once rinsed, they were ready for immediate photography. Simultaneously, another set of cardiac slices was subjected to fixation using 10% neutral formaldehyde. This fixation process was carried out for 6 h to ensure proper preservation of the tissue structure for further analysis.

For image analysis, the caudal side of every slice was chosen, and a pathological graphic analysis system was utilized. In each slice, the infarcted area and the overall area were measured. The infarcted volume for each layer was determined by multiplying the infarcted area of that particular layer by the layer's thickness. The cumulative infarcted volume of all layers constituted the total infarcted volume. Eventually, the proportion of the infarcted area of the myocardium was computed as the quotient of the infarcted area of the myocardium divided by the total left-ventricular area.

### 2.5 Tissue paraffin embedding and sectioning

When preparing cardiac tissue samples for histological examination, the initial step involved carefully excising a suitable quantity of cardiac tissue. This tissue was then gently rinsed with PBS to remove any contaminants. After rinsing, the tissue was immersed in 4% paraformaldehyde for a period of 24–48 h to achieve fixation.

Upon completion of the fixation process, the fixed cardiac tissues underwent a step—by—step dehydration procedure. They were first immersed in 75% ethanol for 4 h, followed by 85% ethanol for 2 h, 90% ethanol for 2 h, 95% ethanol for 60 min,100% ethanol I for 30 min, and finally 100% ethanol II for 30 min.

Once the dehydration was complete, the tissues were transferred to xylene for soaking, which facilitated their transparency. Subsequently, they were placed in a 1:1 mixture of anhydrous ethanol and xylene for 8 min, and then successively in xylene I and xylene II, each for 8 min. Next, the transparent tissues were immersed in paraffin wax for soaking and penetration, spending 60 min each in paraffin I, paraffin II, and paraffin III. Subsequently, the tissue block was placed into a mould containing paraffin, its position was arranged with the cut side down, the plastic mould lid was covered, the paraffin was filled up, and after allowing the paraffin to cool down, the paraffin block was quickly removed. Finally, for slicing, the tissue block was fixed on the slicer, slices with a thickness of 3 μm were cut, these slices were first put into 20% alcohol and then into a 47°C water bath to spread them. After spreading, the slices were patched onto cationic slides, placed in a 64°C oven after patching, and finally put into a slice box after the paraffin wax melted and the slides were removed.

### 2.6 HE staining

During the histological sample preparation, the paraffin sections were initially processed in the baking phase. They were placed in a 64°C constant-temperature oven for one hour. After that, the slides holding the sections were left in the same oven for an additional hour of baking. Subsequently, the dewaxing stage commenced. The slides were submerged in xylene Ⅰ for ten minutes, after which they were transferred to xylene Ⅱ for another ten-minute soak. Following dewaxing, the hydration process began. The slides were sequentially immersed in 100% alcohol Ⅰ for five minutes, 100% alcohol Ⅱ for five minutes, 95% alcohol for five minutes, 80% alcohol for three minutes, and 70% alcohol for two minutes. After that, they were rinsed three times with PBS, with each rinse lasting five minutes.

It came to hematoxylin re-staining, the slides were placed in hematoxylin for five minutes to achieve staining. Then, they were rinsed with distilled water. Next, they were put into an alcohol-hydrochloric acid solution for 10–15 s for differentiation purposes, and finally, immersed in tap water for at least 15 min to allow the color to return to blue.

After the hematoxylin-related procedures, the eosin-staining process was carried out. The slide was immersed in eosin for a duration of 5–10 s and was then given a single wash with distilled water. Subsequently, the dehydration stage commenced. The slide was placed in 95% alcohol for 5–10 s, followed by immersion in 100% alcohol I for five minutes and then 100% alcohol II for another five minutes. Following dehydration, the transparency step was initiated. The slide was first placed in xylene I for ten minutes and then transferred to xylene II for an additional ten-minute period. Finally, the slide was sealed with neutral gum, and the entire specimen on the slide was scanned for further analysis.

### 2.7 Masson staining

Prior to its clinical application, the Weigent iron hematoxylin staining solution was formulated by combining reagent A1 and reagent A2 in an equal 1:1 proportion. This freshly-prepared staining solution was then carefully dropped onto the sections to completely cover them, and the sections were left to be stained for 5–10 min. Once the staining time elapsed, the surplus staining solution was gently rinsed off using distilled water. Immediately after, drops of the acidic ethanol differentiation solution were added, and the differentiation process lasted for 5–15 s. After differentiation, the sections were washed with distilled water for 30 s. Subsequently, in order to enhance the staining effect, the sections underwent a second staining procedure. The Weigent iron hematoxylin staining solution was reapplied, and the sections were stained for another 5–10 min.

Subsequently, the sections were immersed in the Masson bluing solution for 3–5 min to restore the blue color. After that, they were rinsed with distilled water for 30 s to remove not only the bluing solution but also any remaining traces of the previously used staining solutions. Following this, the sections were subjected to staining with the Lichun red magenta staining solution for a period of 5–10 min. During the earlier staining processes, a weak-acid working solution was prepared by mixing distilled water and the weak-acid solution in a 2:1 ratio. Drops of this prepared solution were then added to wash the sections for 30 s. Once the wash was complete, the excess liquid was carefully poured out. Subsequently, the phosphomolybdic acid solution was added, and the sections were left in this solution for 1–2 min. Finally, the sections were washed again with the weak-acid solution for 30 s.

After pouring off the excess liquid once more, the aniline blue staining solution was carefully dropped onto the sections and allowed to act for 1–2 min. Then, drops of the weak acid working solution were added to wash the sections for 30 s. Subsequently, the dehydration process was carried out. The sections were first quickly dehydrated in 95% ethanol for 2–3 s. After that, they were subjected to two rounds of dehydration in anhydrous ethanol, each round lasting 5–10 s. Following dehydration, the transparency step was initiated. The sections were made transparent by being placed in xylene twice, with each treatment lasting 1–2 min. After achieving transparency, the sections were sealed with neutral gum. Then, the entire slide was scanned. Finally, the scanned results were analyzed using ImageJ-pro-plus software. To present the data visually, Prism software was employed to generate histogram statistics (*P* < 0.05).

### 2.8 RNA sequencing and data preprocessing

TRIzol was utilized to isolate and purify total RNA from rats specimens. Subsequently, the quantity of the total RNA was measured with a NanoDrop ND-1000 spectrophotometer, while its integrity was evaluated using a Bioanalyzer 2100 system. Only those samples meeting specific criteria were deemed appropriate for subsequent downstream experiments. These criteria included a concentration greater than 50 ng/µL, an RIN value exceeding 7.0, an optical density ratio of OD 260/280 greater than 1.8, and a total RNA amount of more than 1 µg. Afterwards, Dynabeads Oligo (dT)25-61005was employed to purify Poly(A) RNA from 1 µg of total RNA. This purification process consisted of two rounds. Subsequently, the Magnesium RNA Fragmentation Module was utilized to break the poly(A) RNA into smaller fragments. The fragmentation was carried out at 94°C for a duration of 5–7 min. Following this, the cleaved RNA fragments were reverse-transcribed into cDNA using SuperScript™ II Reverse Transcriptase. Additionally, PCR amplification was executed under the given conditions. First, there was an initial denaturation step at 95°C for 3 min. Then, 8 cycles were conducted, with each cycle involving denaturation at 98°C for 15 s, annealing at 60°C for 15 s, and extension at 72°C for 30 s. Finally, a final extension step at 72°C for 5 min was performed.

The mean insert size of the final cDNA library measured 300 ± 50 base pairs (bp). For sequencing, the Illumina NovaSeq 6,000 platform was employed, operating in the paired-end 150 bp (PE150) sequencing mode. Once the sequencing process was completed, the Fastp software was utilized to eliminate low-quality reads. To evaluate the presence of AT/GC separation, the Phred softwarewas used. Notably, the rat reference gene set along with the annotation files were obtained from the Ensembl database (https://asia.ensembl.org/info/about/species.html).

### 2.9 Identification of the target genes of YQHXCP

YQHXCP was composed of Huangqi, Renshen, Sanqi, Tinglizi, Honghua and Yimucao. The active ingredients and targets of YQHXCP were retrieved from the Traditional Chinese Medicine System Pharmacology Database and Analysis Platform (TCMSP, https://tcmspw.com/tcmsp.php, accessed in January 2024). Screening was performed based on oral bioavailability (OB) ≥ 30% and drug-likeness (DL) ≥ 0.18. Additionally, putative targets were predicted using the SwissTargetPrediction database (http://swisstargetprediction.ch, accessed in January 2024) with “Homo sapiens” selected as the organism. The collected human gene symbols were mapped to rat homologs using the NCBI HomoloGene database (Version 68) for subsequent consistent analysis with the animal model data.

To gain a deeper comprehension of the associations among the components of YQHXCP, its active ingredients, and the target genes of YQHXCP, Cytoscape software (V 3.10.2) (16) was used to construct a network diagram that illustrates the connections among these three elements.

### 2.10 Differential expression analysis

To distinguish the gene expression changes in different intervention groups, we defined the differentially expressed genes identified between the C and I samples as DEGs1, those between the K and C samples as DEGs2, and those between the L and C samples as DEGs3. The DESeq2 package (V 1.42.0) (17) was used to identify differentially expressed genes (DEGs) based on the raw count data from three biological replicates per group (*n* = 10). Read counts were normalized using the internal median of ratios method provided by DESeq2. To control the false discovery rate (FDR) caused by multiple hypothesis testing, the Benjamini-Hochberg procedure was applied. Genes with an adjusted *P*-value (P.adjust) < 0.05 and |log2 fold-change (FC)| > 0.5 were considered significantly differentially expressed. The volcano plots were generated using the ggplot2 package (V 3.5.1) (18). For creating heat maps, the pheatmap package (V 1.0.12) (19) was utilized.

### 2.11 Identification of key target genes and functional enrichment analysis

The intersection genes1 were obtained via up-regulated DEGs1, down-regulated DEGs2, and down-regulated DEGs3 using the ggvenn package (V 1.7.3) (20). The intersection genes2 were obtained via down-regulated DEGs1, up-regulated DEGs2, and up-regulated DEGs3 using the ggvenn package (V 1.7.3). Then, Differentially expressed genes (DEGs) were obtained by combining intersection genes1 and intersection genes2. Finally, the DEGs and YQHXCP target genes were intersected using ggvenn package (V 1.7.3) to obtain key target genes.

The clusterProfiler package (V 4.10.1) (21) was used to conduct Gene Ontology (GO) enrichment analysis on the key target genes. This analysis aimed to evaluate the functions associated with the differentially expressed genes (DEGs) (*P* < 0.05).

The Wilcoxon test was applied to assess the differences in the expression levels of key target genes across all pairwise comparisons among the four groups (*P* < 0.05).

### 2.12 Gene set enrichment analysis (GSEA)

For the purpose of clarifying the biological function of key target genes through GSEA, the following steps were taken. Initially, on all samples within the sequencing data, the Spearman correlation between each key target gene and other genes was analyzed. This was accomplished using the psych package (V 2.2.5) (22). Subsequently, the obtained correlation coefficients were sorted in descending order. Following this, the clusterProfiler package (V 4.10.1) (21) was employed to conduct GSEA. As a reference gene set for this GSEA, the “m5.go.v2024.1.Mm.symbols.gmt” file, sourced from the Molecular Signatures Database (MSigDB) (https://www.gsea-msigdb.org/gsea/msigdb), was utilized (P.adjust < 0.05).

### 2.13 Gene-gene interaction (GGI) network and subcellular localisation analysis

In order to explore the functional state of the key target genes and their related genes, the key target genes were inputted into the GeneMANIA database (https://genemania.org/). The species parameter was configured as rat. After that, a GGI network was established. This network consisted of genes associated with the key target genes and their respective functions.

To uncover the precise organelle locations within the cell where the key target genes exert their functions, the sequences of these key target genes were retrieved from the Gene database (https://www.ncbi.nlm.nih.gov/gene/?term=) on the National Center for Biotechnology Information (NCBI) website. Subsequently, the mRNALocater database (http://bio-bigdata.cn/mRNALocater/) was utilized to predict the subcellular localization of the key target genes.

### 2.14 Construction of regulatory network

To probe transcription factors (TFs) that target regulate key target genes through the regulatory network, key target genes-related TFs were predicted in the Network Analyst database (https://www.networkanalyst.ca/), along with the TFs-key target genes regulatory network was constructed using Cytoscape software (V 3.10.2).

### 2.15 Molecular docking

To assess the binding ability between key target genes and drugs, drugs with the highest OB corresponding to key target genes were selected for docking with these genes. The 3D structures of the active ingredients of such drugs were sourced from the Public Chemical Database (PubChem, https://pubchem.ncbi.nih.gov/). These structures were retrieved and downloaded. Concurrently, the Protein Data Bank (PDB) format files of the 3D structures of key target genes were obtained from the UniProtKB database (https://www.uniprot.org/uniprotkb/). Afterward, molecular docking between the 3D structures of key target genes and the active compounds was carried out using CB-DOCK2. This process aimed to determine the binding energies, which would help in understanding the interaction strength between the key target genes and the drugs. Typically, binding energies less than −1.2 kcal/mol were considered to have satisfactory docking bonding properties, and binding energies less than −5 kcal/mol were considered to have strong binding properties.

### 2.16 Reverse transcription quantitative polymerase chain reaction (RT-qPCR)

The I group, C group, K group and L group cardiac tissue samples were gained from the rat mode. Cardiac tissue samples were obtained from the specimens to conduct RT-qPCR. The research was approved by Ethics Committee of the Third Affiliated Hospital of Zhejiang Chinese Medical University. The expression levels of key target genes were further verified through RT-qPCR. In accordance with the manufacturer's guidelines, total RNA from the cardiac tissue samples was extracted using TRizol. Then, following the manufacturer's instructions, the SweScript First Strand cDNA synthesis kit was utilized to perform the reverse transcription of the total RNA into cDNA.

RT-qPCR was carried out using the 2xUniversal Blue SYBR Green qPCR Master Mix. The primer sequences employed for RT-qPCR are presented in Table 1. GAPDH served as the internal reference gene. The 2−ΔΔCt method was applied to calculate the expression levels of key target genes. Additionally, GraphPad Prism 5 software was utilized to illustrate the differences in the expression of key target genes among the C and I groups, K and C groups, and L and C groups. (*P* < 0.05).

**Table 1:** The key target genes-specific primers (PCR primer sequence).

Primer	GenBank
TOP2a F	TGTGGAGAAGCGTCCAAGTC
TOP2a R	TGACAACTCCATGGTGACCG
CDK1 F	GGAACAGAGAGGGTCCGTTG
CDK1 R	GCACTCCTTCTTCCTCGCTT
E2F2 F	ACGGCGCAACCTACAAAGAG
E2F2 R	GTCTGCGTGTAAAGCGAAGTG
The internal reference gene R-GAPDH F	GGCCGGAGACGAATGGAAATTA
The internal reference gene R-GAPDH R	CCAAATCCGTTCACACCGAC

### 2.17 Statistical analysis

Statistical analysis and data visualization were performed using R software (V 4.2.2) and GraphPad Prism. Continuous variables are expressed as mean ± standard deviation (SD). For comparisons involving more than two groups, a one-way analysis of variance (ANOVA) followed by Tukey's *post-hoc* test was utilized to adjust for multiple comparisons. For transcriptomic data, the Benjamini-Hochberg procedure was applied to adjust *P*-values to control the false discovery rate (FDR) during multiple testing. There were no missing data, and no data points were excluded from the final analyses. Exact *P*-values are reported where applicable. Statistical significance was defined as *P* < 0.05 or an adjusted *P*-value (P.adjust) < 0.05.

## 3 Results

### 3.1 The effects of YQHXCP on cardiac function and myocardial tissue

**Figure 2:**
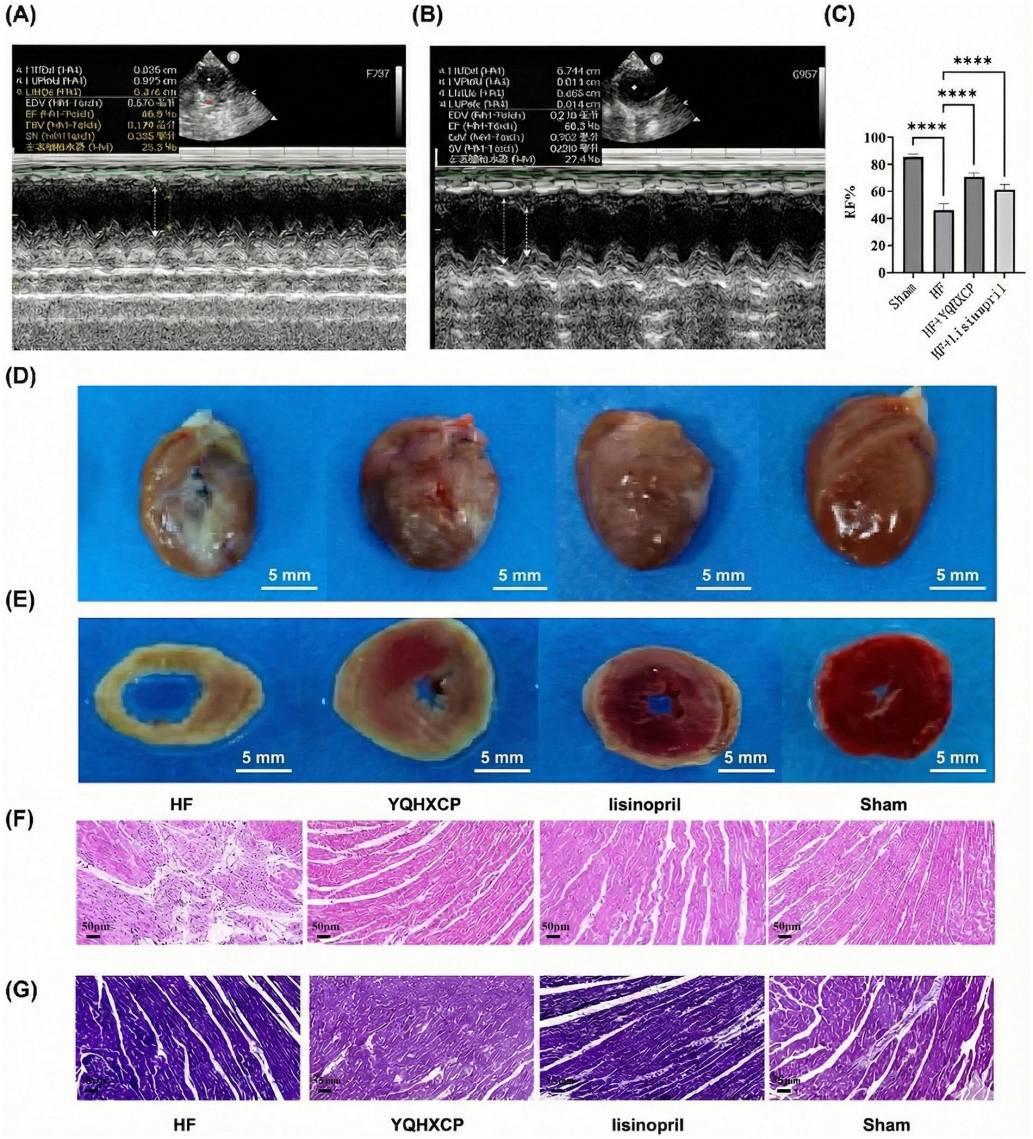
Effects of YQHXCP and lisinopril on cardiac function and myocardial tissue morphology. **(A–C)** After treatment, the EF values in the YQHXCP group (K) and lisinopril group (L) were significantly higher than those in the heart failure group (C) (*P* < 0.05). K: YQHXCP group; L: lisinopril group. **(D)** Gross morphological appearance of cardiac tissue in each group. **(E)** Triphenyl tetrazolium chloride (TTC) staining in each group, where the ischemic area was reduced in K and L groups. **(F)** Hematoxylin and Eosin (HE) staining showing cellular arrangement, with reduced infarct area in K and L groups. **(G)** Masson's trichrome staining highlighting myocardial fibrosis, demonstrating significantly reduced lesions in K and L groups compared to the C group.

After the administration of YQHXCP and lisinopril, doppler echocardiographic detection of EF values in K and L groups were significantly higher than C group (Figures 2A,B). Notably, YQHXCP was more effective in enhancing the EF values (*P* < 0.05) (Figure 2C). Cardiac tissue examination revealed that, compared to I group, the anterior wall of the left ventricle in C group was pale and the heart chambers were enlarged. However, after treatment with YQHXCP and lisinopril, these visual features improved (Figure 2D). These findings demonstrated that both YQHXCP and lisinopril treatments led to an enhancement in the heart's contractile function.

The results of TTC staining revealed that ischemia was aggravated in I group, while the ischemic area was reduced in K and L groups (Figure 2E). This suggests that YQHXCP and Lisinopril improved the myocardial ischemia. HE staining showed that in I group, cardiomyocytes were neatly arranged, with homogeneous morphology and uniform size. In contrast, in C group, cardiomyocytes were markedly reduced, the arrangement of myofibres was disordered, and there was massive infiltration of inflammatory cells. In K and L groups, the infarct area was reduced (Figure 2F). Masson staining indicated that in the C group, the infarcted area exhibited evident myocardial fibrosis. In the infarct-margin area, the cells were arranged in a loose manner, and there was a substantial deposition of collagen fibres in the cell-interstitial space. Compared with the C group, myocardial-tissue lesions were significantly reduced in K and L groups (Figure 2G). The above results indicated that the treatment with YQHXCP and lisinopril could improve cardiac ischemia, reduce cardiac-tissue lesions, and mitigate fibrosis in HF rats.

### 3.2 Identification and functional enrichment of key target genes

**Figure 3:**
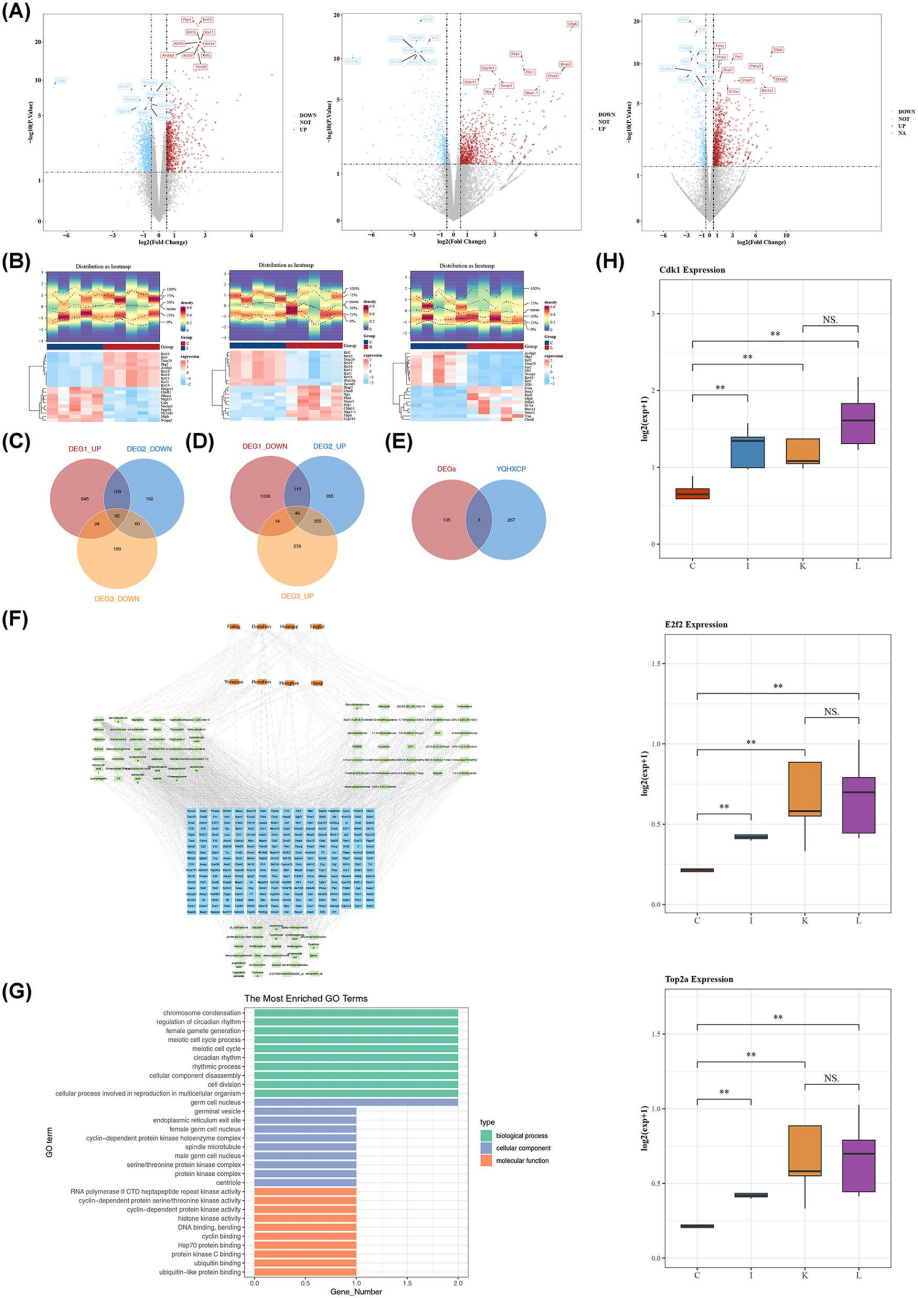
Identification and functional enrichment of key target genes. **(A)** Volcano plots of differentially expressed genes. Left: Heart failure group vs. sham surgery group (DEGs1); Center: YQHXCP group vs. heart failure group (DEGs2); Right: Lisinopril group vs. heart failure group (DEGs3). **(B)** Differentially expressed gene heatmaps corresponding to the comparisons in panel A. **(C)** Venn diagram showing the intersection of DEGs1-upregulated genes and DEGs2/DEGs3-downregulated genes. **(D)** Venn diagram showing the intersection of DEGs1-downregulated genes and DEGs2/DEGs3-upregulated genes. **(E)** Venn diagram showing the intersection of DEGs and YQHXCP to yield the three key target genes. **(F)** Drug-component-mRNA Network illustrating interactions. **(G)** GO enrichment analysis of key target genes. **(H)** Key target gene expression in sham group (I), heart failure group (C), YQHXCP group (K), and lisinopril group (L) samples. The labels for Sham and Heart Failure have been corrected to match the methodology.

From the sequencing data of C and I samples, 2,079 differentially expressed genes1 (DEGs1) were identified. Among them, in C samples, 870 DEGs1 were up-modulated and 1,209 were down-modulated. Based on the sequencing data between K and C samples, 1,319 differentially expressed genes2 (DEGs2) were determined. In K samples, 866 DEGs2 were up-modulated and 453 were down-modulated. For the sequencing data between L and C samples, 1,366 differentially expressed genes3 (DEGs3) were found. In L samples, 991 DEGs3 were up-modulated and 375 were down-modulated (Figures 3A,B).

The 92 intersection genes1 were found through the intersection among up-regulated DEGs1, down-regulated DEGs2 and down-regulated DEGs3 (Figure 3C). The 46 intersection genes2 were found through the intersection among down-regulated DEGs1, up-regulated DEGs2 and up-regulated DEGs3 (Figure 3D). Thereafter, 138 DEGs were obtained for subsequent analyses by intersecting gene1 and intersecting gene2. Then, the 270 YQHXCP target genes were confirmed via the TCMSP and SwissTargetPrediction databases. To identify targets that are both pharmacologically accessible and pathologically critical, we intersected the experimentally observed DEGs with the theoretically predicted YQHXCP targets. This intersection narrowed down the candidate list to 3 key target genes: CDK1, TOP2A, and E2F2, suggesting these genes represent the core molecular nodes where the compound's active ingredients exert their regulatory effects on the disturbed HF network (Figure 3E).

Afterwards, the YQHXCP components-YQHXCP active ingredients-YQHXCP target genes network demonstrated the interactions of them (Figure 3F). For example, there was an interaction among TOP2A, danshen and luteolin, as well as among Cdk1, honghua and quercetin. The 3 key target genes were involved in 218 GO terms, which comprised 182 biological processes (BPs), 18 cellular components (CCs), as well as 18 molecular functions (MFs) (Figure 3G). Specifically, these included chromosome condensation and regulation of circadian rhythm in BP, germ cell nucleus and endoplasmic reticulum exit site in CC, RNA polymerase II CTD heptapeptide repeat kinase activity as well as histone kinase activity in MF.

The Kruskal test showed that the expression magnitudes of the three key target genes differed significantly between I and C samples, between K and C samples, as well as between L and C samples in the sequencing data (*P* < 0.05). Among them, three target genes were significantly down-regulated in the C samples (Figure 3H).

### 3.3 Pathways of three key target genes

**Figure 4:**
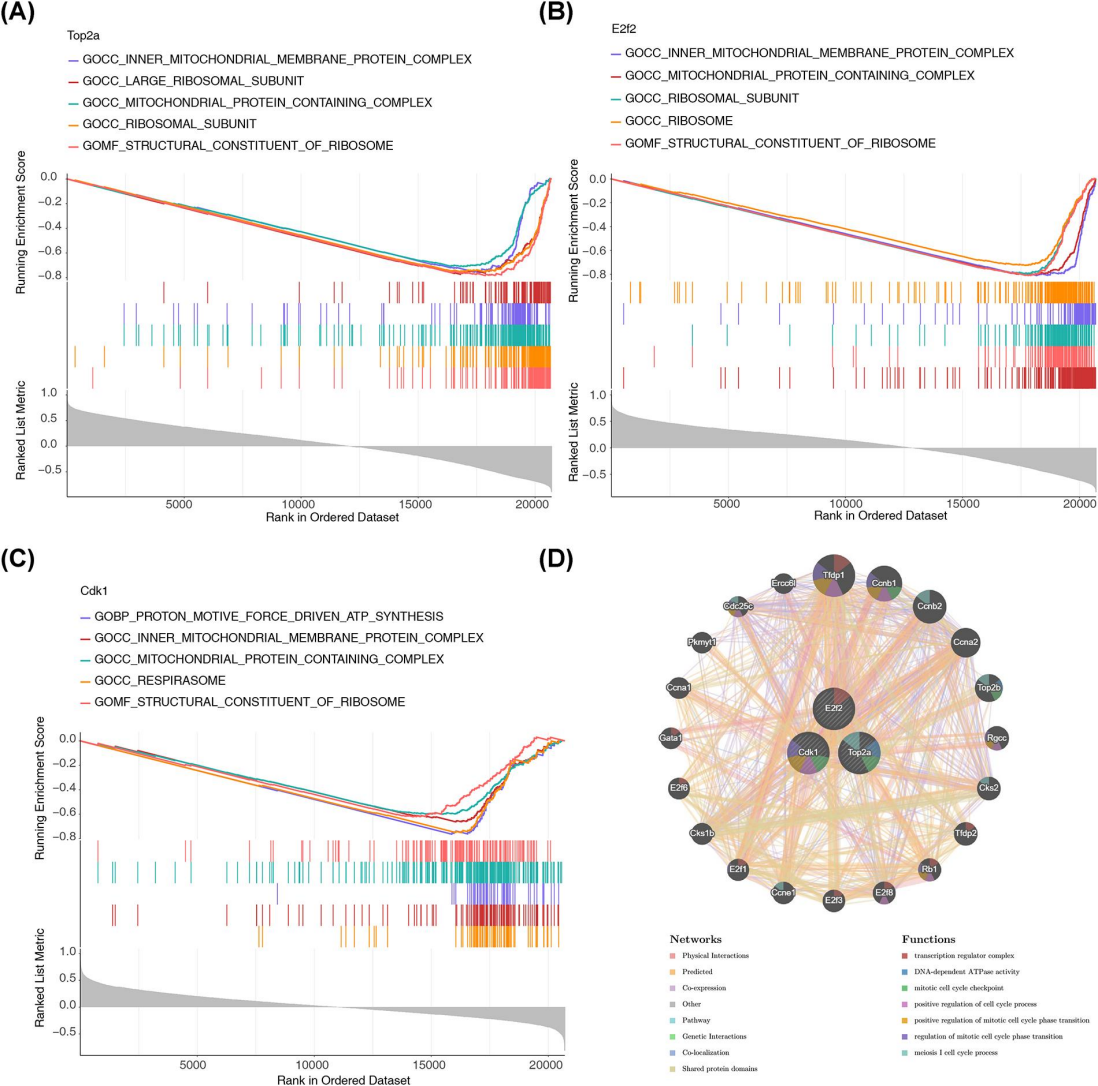
Pathways of three key target genes. **(A–C)** Gene Set Enrichment Analysis (GSEA) running enrichment score plots for Top2a, E2f2, and Cdk1, respectively. Colored lines represent distinct Gene Ontology (GO) terms spanning Cellular Component (GOCC), Molecular Function (GOMF), and Biological Process (GOBP). **(D)** Gene-Gene Interaction (GGI) network results via GeneMANIA.

According to the threshold of *P* < 0.05,1,896 pathways were significantly enriched in association with Top2a, including structural constituent of ribosome, ribosomal subunit, mitochondrial protein containing complex, and others (Figure 4A). Then, the 2,102 pathways were significantly enriched in association with E2f2, including mitochondrial protein containing complex, proton motive force driven ATP, and others (Figure 4B). Subsequently, the 1,586 pathways were significantly enriched in association with Cdk1, including mitochondrial protein containing complex, NADH dehydrogenase complex assembly, and others (Figure 4C). It was suggested that the three key target genes might collectively influence the mitochondrial protein containing complex pathway involved in YQHXCP treatment of HF.

The results from GGI network indicated that 20 genes were linked to three key target genes, which participated in pathways related to transcription regulator complex, DNA-dependent ATPase activity, mitotic cell cycle checkpoint, and so on (Figure 4D). These results revealed that Top2a and Cdk1 might affect YQHXCP treatment of HF via the modulation of 20 genes that mitotic cell cycle checkpoint.

### 3.4 Subcellular localisation and regulatory networks of three key target genes

**Figure 5:**
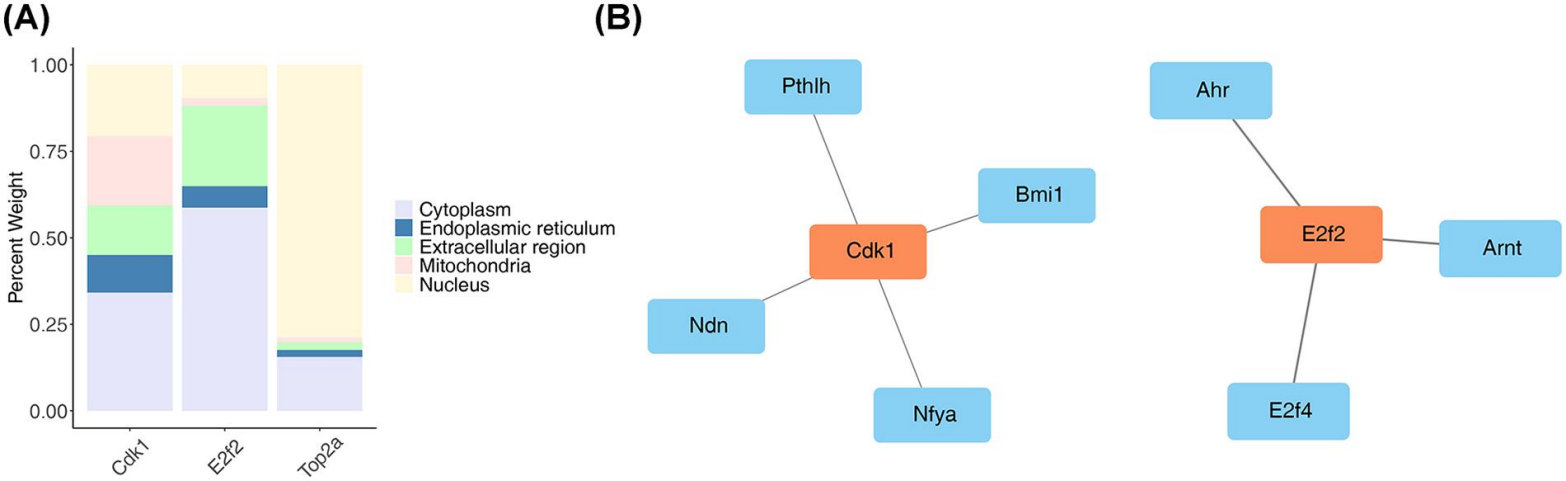
Subcellular localisation and regulatory networks of three key target genes. **(A)** Subcellular localization bar plot indicating Top2a is mainly distributed in the nucleus, while E2f2 and Cdk1 are mainly distributed in the cytoplasm. **(B)** TF-mRNA Network showing predicted transcription factors for Cdk1 and E2f2.

Subcellular localisation showed that Top2a was mainly distributed in the nucleus, E2f2 and Cdk1 were mainly distributed in the cytoplasm (Figure 5A). These results suggested that E2f2 and Cdk1 might influence cytoplasmic involvement in YQHXCP treatment of HF processes.

In total, seven TFs were predicted via three key target genes. Among them, four TFs were predicted via Cdk1, for example, Bmi1, Ndn, and Nfya. Three TFs were predicted via E2f2, for example, Ahr, Arnt, and E2f4. Top2a did not yield any predictions regarding TFs (Figures 5B,C). The findings detailed above indicated that Bmi1 and Ahr potentially exerted an influence on the functions of Cdk1 and E2f2. Additionally, they appeared to contribute to the curative effect of YQHXCP in the treatment of HF.

### 3.5 Molecular docking of three key target genes

**Figure 6:**
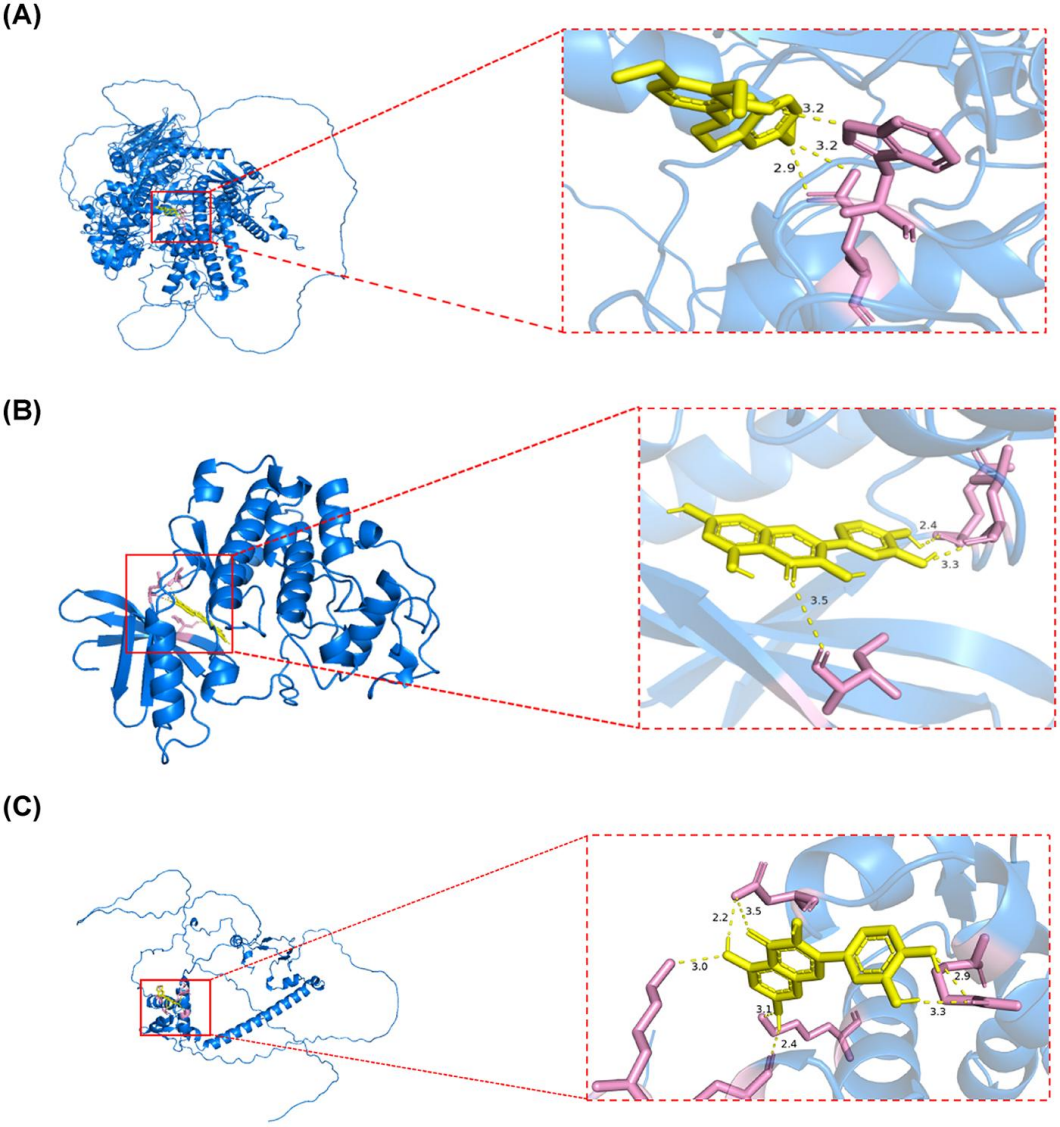
Molecular docking of three key target genes. **(A)** Molecular docking results for Top2a-7-O-Methylisomucronulatol. **(B)** Molecular docking results for Cdk1-quercetin. **(C)** Molecular docking results for E2f2-quercetin. Yellow dashed lines represent hydrogen bonds between the ligand and the protein, with corresponding interaction distances measured in Angstroms **(Å)**.

Molecular docking results demonstrated that 7-O-Methylisomucronulatol had a high binding affinity for Top2a, with a binding free energy of −7.9 kcal/mol (Table 2). In the interaction between 7-O-Methylisomucronulatol and the binding protein Top2a, the lengths of the hydrogen bonds were 3.2 Å and 2.9 Å, respectively (Figure 6A). Quercetin exhibited a strong binding affinity for Cdk1, with a binding free energy of −6.7 kcal/mol (Table 2). The hydrogen-bond lengths between quercetin and the binding protein Cdk1 were 3.5 Å, 3.3 Å, and 2.4 Å, respectively (Figure 6B). Moreover, quercetin also showed a significant binding affinity for E2f2, with a binding free energy of −6.4 kcal/mol (Table 2). The length of hydrogen bond between the quercetin and the binding protein E2f2 were 3.0 Å, 2.2 Å, 3.5 Å, 3.1 Å, 2.4 Å, 3.3 Å and 2.9 Å, respectively (Figure 6C). The aforementioned findings suggested that both Cdk1 and E2f2 were bound with quercetin.

**Table 2:** Docking binding capacity of YQHXCP and key target genes.

Key target genes	Active ingredients	Binding energy
Top2a	7-O-Methylisomucronulatol	−7.9 kcal/mol
Cdk1	Quercetin	−6.7 kcal/mol
E2f2	Quercetin	−6.4 kcal/mol

### 3.6 Validation of key target genes expression

**Figure 7:**
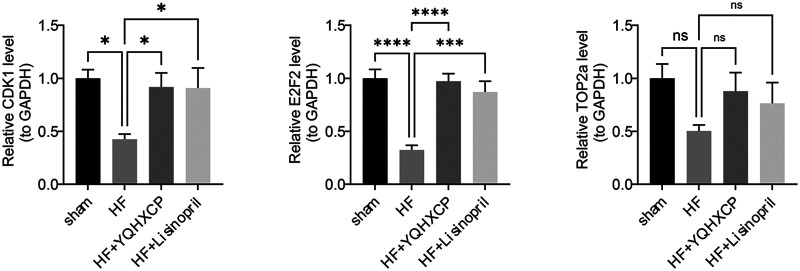
Validation of key target gene expression. The RT-qPCR result of the expression levels of the three target genes (CDK1, E2F2, TOP2a) in each group, normalized to GAPDH. Data are presented showing significant downregulation of Cdk1 and E2f2 in HF (*P* < 0.05), while Top2a was not significantly different. Statistical significance is indicated by asterisks (* *P* < 0.05, *** *P* < 0.001, **** *P* < 0.0001) or ns (not significant).

The RT-qPCR found that the Cdk1 and E2f2 were markedly down-modulated in HF (*P* < 0.05), consistent with the results of Kruskal test, while the Top2a gene was not significantly different between groups (Figure 7). The aforementioned findings suggested that Cdk1 and E2f2 might have been the targets of action of YQHXCP in the treatment of HF patients.

## 4 Discussion

The core pathogenesis of CHF in Traditional Chinese Medicine (TCM) is “deficiency as the root and excess as the manifestation”, meaning that heart qi deficiency constitutes the fundamental condition, while blood stasis and fluid retention are the superficial manifestations. The therapeutic strategy of reinforcing qi and promoting blood circulation (Yiqi Huoxue) directly addresses this pathogenesis (23), and YQHXCP represents a concrete application of this approach (24). To systematically elucidate the modern scientific basis of YQHXCP in treating CHF, this study integrated network pharmacology with cardiac transcriptomic analysis. We first established a rat model of CHF and confirmed that YQHXCP significantly improved cardiac function and attenuated myocardial injury and fibrosis. Subsequently, by cross-analyzing the potential targets of the formula with differentially expressed genes in CHF, we identified three key targets: TOP2A, CDK1, and E2F2. This suggests that YQHXCP may exert its therapeutic effects on CHF through multiple targets and pathways, reflecting the holistic regulatory characteristics of traditional Chinese herbal formulas.

Topoisomerase 2α (TOP2A) participates in the regulation of gene transcription by facilitating the binding of RNA polymerase to the DNA template, thereby promoting the initiation and elongation of transcription (25). By altering the topological structure of DNA, it enables transcription factors and other proteins to better recognize and bind to specific DNA sequences, thus modulating the level of gene expression (26). Mutations or aberrant expression of the TOP2A gene may lead to dysfunction of TOP2A, impairing DNA damage repair mechanisms in cardiomyocytes. Accumulation of DNA damage can activate intracellular apoptotic signaling pathways (27), resulting in increased cardiomyocyte apoptosis. The reduction in cardiomyocyte number directly compromises cardiac contractile function, which, over time, can lead to heart failure (28). The RT-qPCR validation results in this study showed that YQHXCP intervention did not significantly alter the mRNA expression levels of TOP2A in myocardial tissue. Therefore, the role of TOP2A predicted by network pharmacology should be interpreted with caution. It is plausible that YQHXCP regulates TOP2A at the post-translational modification or enzymatic activity level rather than at the transcriptional level. Alternatively, TOP2A may act as an indirect node in the broader interaction network rather than a direct therapeutic target. These results also highlight the robustness of CDK1 and E2F2, which were successfully validated, as more direct potential targets of YQHXCP.

E2F2, a crucial member of the E2F transcription factor family, primarily regulates the transition of the cell cycle from the G1 phase to the S phase, driving the expression of genes involved in DNA replication and cell proliferation (29). In this study, integrated analysis of transcriptomics and network pharmacology identified E2F2 as one of the key targets of YQHXCP in the intervention of CHF. Notably, RT-qPCR validation confirmed that the mRNA expression level of E2F2 was significantly downregulated in the myocardial tissue of CHF model rats, and this downregulation was markedly reversed following YQHXCP treatment. This expression trend is fully consistent with the predictions from bioinformatic analysis, providing strong support for the reliability of E2F2 as an effective target of YQHXCP. We hypothesize that YQHXCP, by upregulating E2F2 expression, may activate downstream gene networks associated with cell cycle progression and DNA synthesis (29). In terminally differentiated cells such as adult mammalian cardiomyocytes, appropriate activation of such programs might improve cardiomyocyte function—possibly by promoting mitochondrial biogenesis, enhancing metabolic capacity, or triggering limited repair mechanisms—thereby counteracting energy depletion and maladaptive responses during CHF progression (30).

Cyclin-dependent kinase1(CKD1) is one of the key kinases that regulate the transition of cells from the G2 phase to the M phase during the cell cycle (31). The activity of CDK1 itself is tightly regulated by multiple factors, including binding to cyclin B, as well as phosphorylation and dephosphorylation modifications (32). In late G2 phase, cyclin B gradually accumulates and binds to CDK1, forming the CDK1-cyclin B complex. This complex is then phosphorylated at Thr14 and Tyr15 residues of CDK1 by kinases such as Wee1, rendering CKK1 inactive (33). When intracellular conditions are suitable for entry into M phase, the phosphatase Cdc25 is activated. It removes the phosphate groups from the Thr14 and Tyr15 residues of CDK1, thereby activating CDK1 and enabling it to exert its regulatory functions on the cell cycle. During the development and progression of heart failure, the normal cell cycle regulation in cardiomyocytes is often disrupted. Studies have shown that the activity and expression levels of CDK1 may be altered, affecting cardiomyocyte proliferation and renewal. For example, in heart failure models induced by excessive pressure overload, CDK1 activity may be suppressed, preventing cardiomyocytes from properly entering the mitotic phase. This impairs the effective replenishment of cardiomyocyte numbers, thereby compromising the heart's capacity for repair and compensation.

GSEA analysis revealed that the above three key target genes were significantly enriched in pathways related to mitochondrial protein complexes. GO enrichment analysis indicated that, in terms of molecular function, these three key genes were significantly enriched in pathways including cyclin-dependent protein serine/threonine kinase activity, cyclin-dependent protein kinase activity, histone kinase activity, DNA binding, and DNA bending. GGI network analysis indicated that the three key targets are co-expressed with 20 genes involved in transcriptional regulation and mitotic checkpoints. As pivotal transcriptional regulators (26, 34), TOP2A and E2F2 are hypothesized to facilitate the transcription of nuclear-encoded mitochondrial proteins. Concurrently, CDK1 and TOP2A may assist cardiomyocytes in managing replicative stress by modulating cell cycle checkpoints (35, 36). These observations align with the GO enrichment results, suggesting that YQHXCP treatment is associated with enhanced mitochondrial function and improved energy metabolism, potentially mediated by the upregulation of these key targets. Therefore, YQHXCP likely enhances mitochondrial function at the transcriptional level by upregulating these targets, thereby improving energy supply in failing myocardium. This provides a potential metabolic mechanism underlying its therapeutic efficacy.

The regulatory network and molecular docking analysis results provide important clues for revealing the multi-level regulatory mechanisms underlying YQHXCP's therapeutic effects on CHF. YQHXCP may modulate the expression of core targets by regulating upstream transcription factors (TFs), thereby amplifying its therapeutic efficacy. E2F2 is predicted to be regulated by E2F4 and the Ahr/Arnt complex. Members of the E2F family, such as E2F4, finely control cell cycle progression and DNA damage response through complex feedback loops (37). In the context of CHF, oxidative stress and metabolic dysregulation are key features, and the aryl hydrocarbon receptor (Ahr), as a critical sensor of environmental stress and metabolic signals, has been associated with cardioprotective effects upon activation (38, 39). Therefore, we hypothesize that active components in YQHXCP may upregulate E2F2 expression by modulating the Ahr signaling pathway or influencing E2F4 activity, ultimately promoting gene transcription programs conducive to cardiomyocyte survival and metabolic adaptation. Furthermore, molecular docking results indicate that quercetin, a core active compound in the formula, exhibits strong binding affinity to both CDK1 and E2F2 proteins. As a well-known natural compound, quercetin has been extensively documented in the literature for its cardioprotective effects, including antioxidant, anti-inflammatory, and anti-myocardial fibrosis properties (40). The findings of this study provide novel mechanistic insights into quercetin's actions: it may directly bind to and modulate the activity of CDK1 and E2F2, thereby coordinately regulating cell cycle checkpoints and transcriptional programs to suppress aberrant cardiomyocyte apoptosis and pathological hypertrophy during heart failure progression.

## 5 Conclusion

This study demonstrates that the YQHXCP exerts therapeutic effects on CHF through a mechanism involving multiple components, multiple targets, and integrated regulation. Integrated bioinformatics analysis identified TOP2A, E2F2, and CDK1 as key targets, which were significantly enriched in critical molecular function pathways such as mitochondrial protein complexes, cell cycle regulation, cyclin-dependent kinase activity, and DNA binding. Regulatory network analysis suggests that these targets may influence transcriptional regulation and cell cycle checkpoints through interactions with related genes and transcription factors. Molecular docking results indicate that potential active compounds in the formula, such as quercetin and 7-O-methylisokirsutic acid, may have strong binding affinity to these targets. Experimental validation confirmed that CDK1 and E2F2 are significantly downregulated in heart failure and can be modulated by YQHXCP treatment, highlighting their potential as therapeutic targets.

Despite these promising findings, this study has several limitations. First, the results are partially based on bioinformatic predictions and public databases, which may contain inherent biases; therefore, the direct physical interactions between the active ingredients and targets require further biophysical verification (e.g., Surface Plasmon Resonance). Second, while we validated mRNA expression levels, protein-level validation (such as Western blotting or immunohistochemistry) is necessary to confirm translational efficiency. Third, this study only evaluated a single therapeutic dosage of YQHXCP calculated based on the clinically equivalent dose. Future comprehensive *in vivo* and *in vitro* studies incorporating multiple dosage gradients are needed to establish a clear dose-response relationship, further confirm these findings, and explore the upstream-downstream regulatory networks of key target genes and core pathways. Finally, the specific downstream signaling cascades activated by Cdk1 and E2f2 warrant further investigation using gene-knockout or overexpression models, thereby laying a solid theoretical foundation for the development of traditional Chinese medicine compound formulations for the treatment of chronic heart failure (CHF).

## Data Availability

The datasets presented in this study can be found in online repositories. The names of the repository/repositories and accession number(s) can be found in the article/Supplementary Material.
